# CD10 Expression in Colorectal Adenoma and Carcinoma and Its Association with the Pathological Prognostic Factors 

**DOI:** 10.30699/ijp.2024.2013354.3185

**Published:** 2024-10-02

**Authors:** Bhavana G R, Clement Wilfred D

**Affiliations:** *Department of Pathology, Ramaiah Medical College, Ramaiah University of Applied Sciences, Bangalore, India*

**Keywords:** Colorectal, Adenocarcinoma, Adenoma, CD10

## Abstract

**Background & Objective:**

Since early detection increases survival rates, colorectal carcinoma (CRC) is a major concern for researchers. CD10 is a cell membrane-bound metalloproteinase involved in carcinogenesis. Studies have associated it with the progression of CRC to advanced stages, metastasis, and venous invasions. We aimed to evaluate the immunohistochemical expression of CD10 in the tumor and stromal cells of colorectal adenoma and CRC and its correlation with the pathological prognostic factors.

**Methods:**

This cross-sectional study was conducted on radical resection specimens of CRC and polypectomy specimens of the colorectal adenomas received for routine histopathological evaluation in the Department of Pathology, Ramaiah Medical College and Hospitals, Bengaluru, from March 2021 to October 2022. Tumor morphology was examined by light microscopy, and CD10 expression was evaluated by immunohistochemistry. Descriptive statistics in terms of percentage and Chi-square test/ Fisher exact tests were used for the statistical analysis.

**Results:**

The study includes 46 cases of adenomas and CRCs each. Stromal CD10 expression was significantly higher in the carcinomas (63.4%) than in the adenomas (41.3%). Proliferative CRCs showed a significantly higher tumoral CD10 expression. The increase in the stromal CD10 expression in CRCs with increasing grades was found to be statistically significant. No significant association was seen between CD10 expression and other factors.

**Conclusion:**

The results indicate a potential role of CD10 in the adenoma-carcinoma sequence. The significant increase in proliferating and high-grade CRCs suggests that CD10 could prove to be a potential biomarker for aggressiveness and also a therapeutic target in CRCs.

## Introduction

Cancer, as one of the most dreaded diseases in the world, is responsible for nearly 30% of deaths from non-communicable diseases in recent years. Colorectal cancer (CRC), which ranks 3rd among the most common cancers in the world, is also the 2nd leading cause of cancer death (1). A marked increase in the incidence of CRC has been observed in recent times, especially in younger age groups of developed countries (2-4). However, it is to be noted that in countries with high human development indexes, such as Australia, Canada, the UK, and the USA, incidence rates of CRC have either decreased or at least stabilized in the past few years. However, this is mainly attributed to sophisticated healthcare systems with well-developed early detection programs (5,6). It can be emphasized that early detection increases survival rates (7,8). Hence, more studies focusing on early detection, pathogenesis, and prognostic markers are a pressing priority in healthcare research (9,10). The tumor cells, or stromal cells, influenced by tumor cells, secrete factors that can disrupt the interaction between normal epithelial cells and stromal cells. Matrix metalloproteinase (MMP) is one such factor and plays an important role in tumor progression (11). A cluster of differentiation 10 (CD10), also known as neprilysin or common acute lymphoblastic leukemia antigen (CALLA), is an MMP and a cell membrane-bound zinc-dependent endopeptidase which is normally expressed in the small bowel and in a variety of cells, including prostate, kidney, adrenal glands, endometrium and lung (12,13). It is expressed in early lymphoid progenitor stages and was first identified on blast cells of ALL (Acute lymphoblastic leukemia) (12). It has also been known to exhibit deranged expression during the development of many tumors like breast carcinoma, where it has been associated with poor prognosis (13). Several studies have detected its expression in CRC in recent years. Investigations have associated this marker with the progression of CRC to more advanced stages, metastasis, and venous invasion in patients (14,15). Further similar studies are warranted to validate the association. Here, we aim to study the expression of CD10 in CRC, which appears to be useful from a diagnostic point of view and can potentially be a prognostic marker.

## Materials and Methods

This cross-sectional study was conducted on radical resection specimens of colorectal carcinoma and polypectomy specimens of colorectal adenomas received in the department of pathology for routine histopathological evaluation from the departments of surgery, surgical oncology and gastroenterology, Ramaiah Medical College and Hospitals, Bengaluru, Karnataka, India. A total of 92 cases of colonic neoplasms were evaluated between March 2021 and October 2022 after obtaining informed consent from the patients. Out of these, 46 cases were adenomas, and 46 cases were adenocarcinomas.

Radical resection specimens of carcinomas and polypectomy specimens of adenomas from patients of ≥18 years were included in the study. Cases where only biopsy, endoscopic mucosal resection, or polypectomy were performed for carcinomas, and those with extensive tumor necrosis without sufficient viable tumor cells were excluded. 

### Histologic Examination

The polypectomy specimens and resection specimens of CRCs were received in the Pathology Department in 10% formalin. In every case, the standard protocol for surgical grossing of resected specimens was followed. For CRCs, the samples were processed as per standard protocol; paraffin-embedded tissue blocks were cut and stained by hematoxylin and eosin (H & E), which were studied and staged according to American Joint Committee on Cancer, 8th edition, 2017. For adenomas, the H&E-stained slides were evaluated for low and high-grade dysplasia and classified accordingly.

### Processing for Immunohistochemistry

 Immunohistochemical detection of CD10 protein was done on sections cut from paraffin blocks of tumor tissue and appropriate control tissue taken on a glass slide coated with an adhesive (poly L lysine). The technique for IHC included antigen retrieval in citrate buffer in a microwave oven, blocking endogenous peroxidase with 3% hydrogen peroxide, incubating with primary mouse monoclonal antibodies against CD10 protein, linking with rabbit anti-mouse secondary antibody, enzyme labeling with streptavidin-horseradish peroxidase, developing chromogen with diaminobenzidine (DAB) and counterstaining with hematoxylin. Positive CD10 expression in tumor epithelial cells (tCD10) was defined as the presence of fine to coarse cytoplasmic and/or membranous staining in 10% or more of cells (11,16). The intensity of stromal CD10 expression (sCD10) was graded according to a 4-point scale based on the percentage of positive cells: 0 (negative; < 10% positive cells), 1+ (10% to 25% of positive cells), 2+ (26% to 50% of positive cells) and 3+ (>50% of positive cells). For statistical analysis, the cases were divided into negative groups (grades 0 and 1) and positive groups (grades 2 and 3) (11,16).

### Statistical Analysis

Descriptive statistics of CD10 expression were analyzed and summarized in terms of percentage. The Chi-square test was used to find the association between CD10 expression and histological grade, depth of tumor invasion, nodal metastasis, and stage. Differences in the proportion of CD10 expression between adenomas and CRCs were tested for statistical significance by chi-square test/ Fisher exact test. 

## Results

The present study comprised 46 cases of adenoma and 46 cases of carcinoma. The mean patient age of adenoma cases was 55.5 years, with majority of the cases occurring in the 6th decade and a male:female ratio of 1.70. Out of the 46 adenoma cases, 37 were of low-grade dysplasia, and 13 were of high-grade dysplasia. They exhibited various histopathological patterns, such as tubular (82.6%), villous (2.17%), tubulovillous (10.87%), and sessile serrated (4.36%). The carcinoma cases had a mean age of 55.78 years with a male:female ratio of 1.55. Other clinicopathological features of the carcinoma cases are depicted in Table 1. Although a higher sCD10 and tCD10 expression were observed in high-grade adenomas than in low-grade adenomas, it was not statistically significant. The expression of sCD10 and tCD10 expression was found to be higher in the carcinoma group as compared to the adenoma group. sCD10 expression of 41.3% in adenomas increased to 63.4% in carcinomas, which was statistically significant (*P*=0.037), as shown in Table 2. The correlation of CD10 expressions with various clinicopathological factors is depicted in Table 3. CD10 expression with respect to age and sex was not statistically significant. Proliferative CRCs showed a significantly (*P*=0.034) higher tCD10 expression (53.57%) compared to the non-proliferative CRCs (22.22%). Expression of sCD10 and tCD10 expression showed no significant association with pT stage. However, an increase in sCD10 expression was seen with the advancing pT stage. sCD10 expression increased significantly with the increasing grades of CRCs (*P*=0.002). No significant association was seen with tCD10 expression in relation to the histological grade or lymph node status. However, a higher sCD10 and tCD10 expression were observed in the cases with positive lymph node status. Although the findings were not statistically significant, advanced-stage CRCs (Stage III and IV) had higher sCD10 and tCD10 expression than early-stage CRCs (Stage I and II).

**Table 1:** Histopathological features of the carcinomas

Features		Frequency (Percentage)
Gross appearance	Non-proliferative	28 (60.87%)
	Proliferative	18 (39.13%)
Histological type	Adenocarcinoma NOS	41 (89.13%)
	Mucinous Adenocarcinoma	3 (6.52%)
	Signet ring cell carcinoma	2 (4.35%)
pT stage	pT2	17 (36.95%)
	pT3	22 (47.83%)
	pT4	7 (15.22%)
Histological type	Adenocarcinoma	41 (89.13%)
	Mucinous adenocarcinoma	3 (6.52%)
	Signet ring cell carcinoma	2 (4.35%)
Histological grade	Grade I	15 (32.61%)
	Grade II	20 (43.48%)
	Grade III	11 (23.91%)
Lymph node status	Positive	23 (50%)
	Negative	23 (50%)
TNM Stage	Stage I	11 (23.91%)
	Stage II	11 (23.91%)
	Stage III	20 (43.48%)
	Stage IV	4 (8.70%)

**Table 2:** Comparison of sCD10 and tCD10 expression between the adenomas and CRCs

	Number of cases	Positive, n (%)	
	**n**	**sCD10**	**tCD10**
Adenoma	46	19 (41.3%)*	12 (26.1%)
Carcinoma	46	29 (63.0%)*	19 (41.3%)

**Table 3:** Expression of sCD10 and tCD10 in relation to the various pathological prognostic factors

		Number of cases	Positive, n (%)	
		**n**	**sCD10**	**tCD10**
Age	**≤45**	12	7 (58.3%)	6 (50%)
	**>45**	34	22 (64.7%)	19 (41.3%)
Sex	**Male**	28	18 (64.3%)	10 (35.7%)
	**Female**	18	11 (61.1%)	9 (50.0%)
Gross appearance	**Non-proliferative**	18	11 (61.1%)	4 (22.22%)*
	**Proliferative**	28	18 (64.3%)	15 (53.57%)*
Depth of invasion	**pT2**	17	10 (58.8%)	7 (41.2%)
	**pT3**	22	14 (63.6%)	10 (45.5%)
	**pT4**	7	5 (71.4%)	2 (28.6%)
Grade	**Grade I**	11	4 (26.7%)*	8 (53.3%)
	**Grade II**	4	16 (80.0%)*	12 (60.0%)
	**Grade III**	2	9 (81.8%)*	8 (72.7%)
Lymph node status	**Positive**	23	13 (56.5%)	9 (39.1%)
	**Negative**	23	16 (69.6%)	10 (43.5%)
Stage	**Early stage**	23	12 (56.5%)	8 (39.1%)
	**Advance stage**	23	17 (69.6%)	11 (43.5%)

*P<0.05

**Table 4:** Comparison of CD10 expression and it’s prognostic value in the various studies

Studies	sCD10 expression	tCD10 expression	Prognostic value	*P*
Jang * et al. * (2)	88%	44%	CD10 expression was found to be higher in CRC as compared to precursor lesions	<0.05
Magadhi * et al. * (4)	49.15%	-	No statistically significant correlation with any prognostic factors	>0.05
Khairy * et al. * (6)	57%	80%	Higher tCD10 expression from low-grade to high-grade adenoma and carcinoma	0.02
Ogawa * et al. * (9)	79.4%	-	Higher sCD10 expression in invasive lesions	<0.0001
Yao * et al. * (12)	-	31.58%	CD10 expression was significantly higher in CRCs with liver metastasis	<0.01
Hirano * et al. * (13)	-	59.41%	Higher CD10 expression was seen in CRCs with high-grade atypia than low-grade atypia	0.01
Roposo * et al. * (14)	88%	88%	Higher tCD10 expression in CRCs with positive lymph node status	0.001
Bernescu * et al. * (15)	-	22.51%	CD10 was not significantly associated with lymph node metastasis	0.33
Current study	63%	41.3%	sCD10 expression is significantly higher in carcinoma than in adenoma	0.037

*P<0.05

## Discussion

The current study, the CD10 marker was targeted for immunohistochemical analysis to determine its role in cancer progression and prognosis. It plays an important role in carcinogenesis by releasing bioactive molecules that stimulate invasion, inhibition of apoptosis, extracellular matrix degradation, immune response modulation, and promotion of angiogenesis (16-18). Important pathological prognostic factors determined the correlation of CD10 expression (19-21). In the present study, both sCD10 and tCD10 expression was found to be higher in the high-grade adenomas as compared to the low-grade adenomas, and further, the expression was seen to be highest in the adenocarcinoma group. The significant increase observed in sCD10 expression from adenoma to carcinoma suggests that CD10 has an important role in colorectal tumorigenesis and the transition sequence from adenoma to adenocarcinoma. This result is in concordance with that of Jang* et al.*, Magadhi* et al.*, Wang* et al.*, Khairy* et al.*, Iwase* et al.*, Ogawa* et al.*, and Koga* et al.*, who reported CD10 expression more frequently in invasive phenotype rather than adenomas (11,13,16,22-25). This supports the involvement of CD10 in progression and carcinogenesis of colorectal carcinoma. In present study the correlation of sCD10 and tCD10 expression in age groups of people ≤45 years of age and people >45 years of age were not statistically significant. Khanh* et al. *in 2011 and Khairy* et al. *in 2015 also observed no significant correlation of CD10 expression with age of the patients in their studies (16,26). In present study, the sCD10 and tCD10 expression was not found to be significantly correlating with gender which was found to be in concordance with the findings by Khairy* et al. *and was in contrast to the findings by Jang* et al. *(11,16). However, the sCD10 expression was found to be higher in males than females in the present study, and it was in agreement with the findings of Jang* et al.*, Khairy* et al.*, and Khanh* et al.*, whereas findings by Iwase* et al.* in contrast to others (11, 16, 23, 26). The tCD10 expression was lower in males than females, which was inconsistent with the findings of other studies (11, 16, 26, 27). It is probably due to differences in sample size and Male:Female ratio in their study group. Both sCD10 and tCD10 expression were higher in proliferative carcinomas than non-proliferative ones. The increase in tCD10 expression among the proliferative carcinomas compared to non-proliferative carcinomas was found to be statistically significant in the present study, which was in concordance with findings by Hirano* et al.*, Oliveira* et al.* and Foda* et al.*, who also observed similar results (14,28,29). In contrast, Koga* et al. *found higher tCD10 expression in non-proliferative than proliferative carcinomas (25). This difference in findings might be due to differences in sample sizes and differences in proportions of proliferative and non-proliferative CRCs in the study. sCD10 expression was found to increase gradually as the depth of the tumor progressed. Magadhi* et al.*, Khairy* et al., *and Khanh* et al. *showed similar findings in their studies (13,16,26). tCD10 expression was found to be low in pT4 as compared to pT2 and pT3 cases in the present study. Jang* et al.*, Khanh* et al. *and Khairy* et al. *observed a decreasing expression of tCD10 with increasing grades. These findings may suggest a role of tCD10 in invasive potential in early CRC. However, there was no statistically significant association of sCD10 and tCD10 expression with depth of invasion (pT stage). In present study, we observed an increase in both sCD10 and tCD10 expression in cases with lymph node metastasis which was not statistically significant. But these findings were similar to the studies conducted by Jang et al, Roposo* et al. *and also Khairy* et al. *(11,16,30). In contrast, Magadhi* et al.*, Bernescu* et al.*, Hirano* et al.*, and Khanh* et al. *found an inverse relationship between CD10 expression and lymph node metastasis (13,26,28,31). This discrepancy may have resulted due to difference in sample sizes and also stages of carcinomas in study group as vascular invasion and lymph node metastasis are generally less frequent in the early colorectal carcinomas. Oliveira* et al. *also did not find statistical significance between CD10 expression and the presence of lymph node metastasis (14). The current study observed an increase in sCD10 expression and a decrease in tCD10 expression as the histological grade progressed from Grade I to Grade III. The findings agreed with most of the previous studies (11,16,26,27,30,31). Also, the increase in sCD10 expression was found to be statistically significant in the current study, unlike the other previous studies. This discrepancy might be due to the difference in sample number and a low number of poorly differentiated (grade III) carcinomas in other studies. However, the decrease in tCD10 expression when increasing grades was not found to be statistically significant in the current study, while it was found to be significant in the study by Khanh* et al. *(26). In the current study, both sCD10 and tCD10 expression were higher in advanced stages (Stage III and IV) as compared to early stages (Stage I and II). No statistical significance was seen in the findings obtained. The findings with respect to sCD10 were not in agreement with those of Khairy* et al. *and Khanh* et al.*, who also did not find a statistically significant correlation (16, 26). Oliveira* et al.*, in their study in 2012*,* also did not find any statistically significant difference in expression of CD10 in relation to CRCs of various stages (14).

## Conclusion

The increase in CD10 expression from low-grade adenoma to high-grade adenoma and, its maximal expression in the adenocarcinoma group, highlights the important role of CD10 in the progression through different stages of the adenoma-carcinoma sequence. A significantly higher tCD10 expression in proliferative CRCs and a significant increase of sCD10 expression through advancing histological grades of CRCs may indicate association of CD10 expression with the malignant behavior of CRCs. Although not statistically significant, an increased expression of sCD10 in the cases with a higher extent of local invasion (pT stage), lymph node metastasis, and advanced TNM stage suggests involvement of sCD10 in invasive potential in CRC. Hence, CD10 could be a new biomarker for aggressiveness and prognostic information. It can also be an attractive and potential therapeutic target that could obtain maximal targeting of cancerous and precancerous tissues with minimal damage to the surrounding normal mucosa. However, further studies with larger sample sizes and survival data are warranted to unequivocally establish the role of CD10 as a therapeutic and prognostic marker in CRC. 

## References

[1] Aceto GM, Catalano T, Curia MC (2020). Molecular Aspects of Colorectal Adenomas: The Interplay among Microenvironment, Oxidative Stress, and Predisposition. BioMed Res Int.

[2] Laskar RS, Talukdar FR, Mondal R, Kannan R, Gosh SK (2014). High frequency of young age rectal cancer in a tertiary care centre of southern Assam, North East India. Indian J Med Res.

[3] Enwerem N, Cho MY, Demb J, Earles A, Heskett KM, Liu L (2021). Systematic Review of Prevalence, Risk Factors, and Risk for Metachronous Advanced Neoplasia in Patients With Young Onset Colorectal Adenoma. Clin Gastroenterol and Hepatol.

[4] Wallace K, Grau MV, Ahnen D, Snover DC, Robertson DJ, Mahnke D et al (2009). The association of lifestyle and dietary factors with the risk for serrated polyps of the colorectum. Cancer Epidemiol Biomarkers Prev.

[5] Senore C, Segnan N, Gunter M, Wild CP, Weiderpass E, Stewart BW (2020). Colorectal cancer. World Cancer Report: Cancer Research for Cancer Prevention.

[6] Gullickson C, Goodman M, JokoFru YW, Gnangnon FHR, N'Da G, Woldegeorgis MA (2021). Colorectal cancer survival in sub-Saharan Africa by age, stage at diagnosis and Human Development Index: A population-based registry study. Int J Cancer.

[7] Chang JJ, Chien CH, Chen SW, Chen LW, Liu CJ, Yen CL (2020). Long term outcomes of colon polyps with high grade dysplasia following endoscopic resection. BMC Gastroenterol.

[8] Guo W, Zheng W, Li JY, Chen JS, Blot WJ (1993). Correlations of colon cancer mortality with dietary factors, serum markers and schistosomiasis in China. Nutr Cancer.

[9] Ahmadi O, Stringer MD, Black MA, McCall JL (2015). Clinicopathological factors influencing lymph node yield in colorectal cancer and impact on survival: analysis of New Zealand Cancer Registry data. J Surg Oncol.

[10] Leslie A, Carey FA, Pratt NR, Steele RJ (2002). The colorectal adenoma-carcinoma sequence. Br J Surg.

[11] Jang TJ, Park JB, Lee JI (2013). The expression of CD10 and CD15 is Progressively increased during Colorectal Cancer Development. Korean J Pathol.

[12] Mishra D, Singh S, Narayan G Role of B cell development marker CD10 in Cancer Progression and Prognosis. Mol Biol Int.

[13] Magadhi M, Panigrahi R, Rath J (2019). Stromal expression of CD10 in Colorectal Carcinoma and its Correlation with Lymph Node Metastasis and Other Prognostic Factors. JCDR.

[14] Oliveira LA, Neto RA, Netto GJ, Sznirer M, Fernandes LC, Lima FO (2012). Tissue expression of CD10 protein in colorectal carcinoma: correlation with the anatomopathological features of the tumour and with lymph node and liver metastases. J Coloproctol.

[15] Zlatian OM, Comănescu MV, Roşu AF, Roşu L, Cruce M, Găman AE (2015). Histochemical and immunohistochemical evidence of tumor heterogeneity in colorectal cancer. Rom J Morphol Embryol.

[16] Khairy RA, Tabak SA, Mahmoud SA, Hamed ATAE (2015). CD10 Expression in Colorectal Carcinoma and Premalignant lesions. Academic J Cancer Res.

[17] Zhu Y, Zheng JJ, Yang F, Nie QQ, Zhu ZL, Deng H (2016). Expression of CD10 in cancer associated fibroblasts and its effect on initiation and progression of colorectal carcinoma. Chin J Pathol.

[18] Żurawski J, Talarska P, de Mezer M (2022). Evaluation of CD10 expression as a diagnostic marker for colorectal cancer. Gastroenterol Hepatol Bed Bench.

[19] Bagante F, Spolverato G, Beal E, Merath K, Chen Q, Akgül O (2018). Impact of histological subtype on the prognosis of patients undergoing surgery for colon cancer. J Surg Oncol.

[20] Vasile L, Olaru A, Munteanu M, Pleşea IE, Surlin V, Tudoraşcu C (2012). Prognosis of colorectal cancer: clinical, pathological and therapeutic correlation. Rom J Morphol Embryol.

[21] Nabi U, Nagi AH, Riaz S, Sami W (2010). Morphological Evaluation of Colorectal Carcinoma with Grading Staging and Histological types. J Pak Med Assoc.

[22] Wang J, ElMasry N, Talbot I, Tomlinson I, Alison MR, ElBahrawy M (2013). Expression profiling of Proliferation and Apoptotic Markers along the Adenoma-Carcinoma sequence in Familial Adenomatous Polyposis patients. Gastroenterol Res Pract.

[23] Iwase T, Kushima R, Mukaisho K, Mitsufuji S, Okanoue T, Hattori T (2005). Overexpression of CD10 and reduced MUC2 expression correlate with the development and progression of colorectal neoplasms. Pathol Res Pract.

[24] Ogawa H, Iwaya K, Izumi M, Kuroda M, Serizawa H, Koyanagi Y (2002). Expression of CD10 by stromal cells during colorectal tumor development. Hum Pathol.

[25] Koga Y, Yao T, Hirahashi M, Kumashiro Y, Ohji Y, Yamada T (2008). Flat adenoma-carcinoma sequence with high-malignancy potential as demonstrated by CD10 and beta catenin expression: A different pathway from the polypoid adenoma-carcinoma sequence. Histopathology.

[26] Khanh DT, Mekata E, Mukaisho K, Sugihara H, Shimizu T, Shiomi H (2011). Prognostic role of CD10+ myeloid cells in association with tumor budding at the invasion front of colorectal cancer. Cancer Sci.

[27] Yao T, Tsutsumi S, Akaiwa Y, Takata M, Nishiyama K, Kabashima A, Tsuneyoshi M (2001). Phenotypic expression of colorectal adenocarcinomas with reference to tumor development and biological behavior. Jpn J Cancer Res.

[28] Hirano K, Nimura S, Mizoguchi M, Hamada Y, Yamashita Y, Iwasaki H (2012). Early colorectal carcinomas: CD10 expression, mucin phenotype and submucosal invasion. Pathol Int.

[29] Foda AARM, Alamer HA, Ikram N, Helali HA, Fayad FS, Hussain SW (2022). Expression of CD10 and CD15 in colorectal mucinous and signet ring adenocarcinomas and its relation to clinicopathological features and prognosis. Cancer biomarkers.

[30] Raposo TP, Comes MS, Idowu A, Agit B, Hassall J, Fadhil W (2018). CD10 inhibits cell motility but expression is associated with advanced stage disease in colorectal cancer. Exp Mol Pathol.

[31] Bernescu I, Reichstein AC, Luchtefeld M, Ogilvie JW (2016). Does CD10 Expression Predict Lymph Node Metastasis in Colorectal Cancer?. Dis Colon Rectum.

